# Myroides cellulitis and bacteremia: A case report

**DOI:** 10.1016/j.idcr.2021.e01061

**Published:** 2021-03-10

**Authors:** William A. Beathard, Aaron Pickering, Micah Jacobs

**Affiliations:** University of Pittsburgh Medical Center (UPMC) St. Margaret, 815 Freeport Rd, Pittsburgh, PA, 15215, United States

**Keywords:** Myroides, Cellulitis, Bacteremia, Antibiotic resistance

## Abstract

Formerly classified under the genus *Flavobacterium, Myroides* species are common gram-negative, environmental bacterium ubiquitous in soil and water. While infrequent, infections of human hosts can result in devastating consequences due the bacteria’s intrinsic multidrug resistance, particularly in those who are immunocompromised. The pathogenicity and mechanisms for resistance remain poorly understood at this time. The case presented in this report details *Myroides* bacteremia secondary to a soft tissue infection of the lower extremities and adds to the 60 documented infections to date, of which 15 were also characterized by a similar infection.

## Introduction

*Flavobacterium*, a genus of bacterium characterized as gram negative, nonmotile, yellow-pigmented, nonsporulating rods that produce a robust, fruity fragrance on nutrient agar was amended to exclude the reclassified genus *Myroides* in 1996 [1,2]. *Myroides odoratum* and *odoratimimus* are two clinically significant species inclusive of the genus are abundantly present environmentally soil and water samples. These bacterial species were originally isolated in feces from patients suffering from typhoid fever and acute gastroenteritis. Review of the literature demonstrates isolation of *Myroides* spp. from urine, blood, respiratory secretions and wound samples from a variety of infections ranging from simple urinary tract infection to more severe conditions such as necrotizing fasciitis [[3], [4], [5]]. The pathogenicity of *Myroides* spp. in human hosts continue to be under documented, however this bacterium’s opportunistic nature lends credence to critical infections in immunocompromised patients [3,[6], [7], [8]]. *Myroides* spp. are acknowledged as having broad, intrinsic antimicrobial resistance profiles and as reported in a single case documenting a M*. odoratimimus* infection of a recurrent calcaneal ulcer in an immunocompromised host, have the propensity to develop biofilms [2,9,10]. This report documents a case of *Myroides* bacteremia secondary to a soft tissue infection of the lower extremities susceptible to meropenem and ciprofloxacin from antibiotics that were tested. Medical literature on documented *Myroides* spp. infections is also reviewed.

## Case presentation

An 88-year-old male with a past medical history significant for persistent atrial fibrillation not on chronic anticoagulation, type 2 diabetes mellitus (most recent HbA1c 6.2 % on 6/26/2020), chronic diastolic heart failure, hypertension, hyperlipidemia, chronic kidney disease stage III, coronary artery disease, and chronic lower extremity edema presented with bilateral extremity swelling and redness of unknown duration, left extremity worse than the right. It is noted that the patient has ambulatory dysfunction and suffers from frequent falls, the most recent sustained approximately one week prior to admission. The family reports a one-week duration of ulceration, edema, and redness in bilateral lower extremities, as well as poor adherence with compression garment therapy. The patient was evaluated by his primary care physician 2 days prior to admission and was diagnosed with cellulitis of the right lower extremity. A 5-day course of oral cephalexin 250 mg 4 times daily was prescribed at that time.

Upon presentation to the hospital, the patient was afebrile and initial vital signs were within normal limits with the exception of ECG notable for atrial flutter with variable AV block in the emergency room. Clinically relevant laboratory studies revealed evidence of an acute kidney injury with a blood urea nitrogen (BUN) and serum creatinine (SCr) of 104 mg/dL and 1.97 mg/dL respectively (baseline SCr of ∼1.6 mg/dL). Physical examination revealed significant bilateral lower extremity edema, warmth and erythematous bilateral lower legs involving the feet with multiple areas of scabbing concerning for cellulitis. Onychomycosis was present with notable scaling between the toes with dried blood.

Infectious workup was significant for an elevated lactate of 3.0 mMol/L and a C-reactive protein of 31.0 mg/dL (normal range 0.0−0.9 mg/dL) in the absence of leukocytosis. The patient was empirically initiated on intravenous vancomycin and received a single 1250 mg dose in the ED in addition to cefepime (1 g every 12 h) for broad-spectrum coverage of cellulitis refractory to oral antibiotics. Blood cultures taken in the ED on hospital day 0 from a peripheral intravenous access site at the right antecubital space grew gram negative rods from the aerobic bottle later speciated by Vitek® 2 system as *Myroides* spp. on day 5 of hospital stay. Growth was confirmed by a second set of blood cultures taken from the right hand obtained on the hospital floor on day 0 also turning positive for *Myroides* spp. on hospital day 5. Susceptibility data revealed multidrug resistance. The isolate demonstrated sensitivity to only ciprofloxacin and meropenem of antibiotics tested (Table 1). Bacterial identification resulted in antimicrobial switch to meropenem 1 g every 8 h on day 4. Leukocytosis clinically peaked on day 7 of admission (12,900 cells/ccm with 66 % neutrophils). Repeat blood cultures were drawn on the floor (on day 4) reflected 5 days of no growth on hospital day 9. Upon clinical resolution of symptoms, the patient was stable for discharge on hospital day 14. The patient was transitioned to oral ciprofloxacin 500 mg twice daily for 6 days at discharge to complete 14 total days of effective antibiotic therapy.

**Table 1:** Culture and Sensitivities [all resistant antibiotics listed in RED font].

Specimen Desc: Peripheral blood	Gram Stain: Gram negative rods from aerobic bottle at 12 h	Culture: *Myroides* spp.
	MIC (mcg/mL)	MIC Interpretation
Amikcain	> = 64	Resistant
Cefazolin	> = 64	Resistant
Cefepime	16	Intermediate
Ceftazidime	> = 64	Resistant
Ceftriaxone	> = 64	Resistant
Ciprofloxacin	1	Sensitive
Gentamicin	> = 16	Resistant
Meropenem	4	Sensitive
Piperacillin/Tazobactam	64	Intermediate
Sulfa/Trimethoprim	> = 320	Resistant

## Discussion

*Myroides* spp., a genus previously classified as *Flavobacterium*, are opportunistic, environmental, gram negative organisms that while infrequently infecting humans, can cause life threatening infections particularly in immunosuppressed persons. A 2019 case report authored by LaVergne and colleagues chronicles a total of just 60 reported infections caused by *Myroides* spp. as listed in the U.S. National Institutes of Health’s National Library of Medicine [11]. Of the 60-total cases, the paper cites 15 cases involving skin and soft tissue infections with 6 of which progressing to bacteremia. A sample of confirmed *Myroides* spp. skin and soft tissues infections illuminates prominent clinical similarities to the present case with respect to the course of infection and multidrug resistance.

A review of the 6 infections involving bacteremia and a report documented after LaVergne’s paper demonstrated patients with ages ranging from 49 to 74 years of age, of which 4 were noted to have immunocompromising underlying conditions [3,[11], [12], [13], [14], [15], [16], [17]]. Of importance, with the exception of diet-controlled type 2 diabetes mellitus (most recent HbA1c 6.2 % on 6/26/2020, HbA1c < 7% for past 4 years), the present case occurred in a patient devoid of major immunocompromising conditions such as malignancy or chronic steroid use. The patient’s advanced age of 88 may have been a contributing immunocompromising condition and led to some degree of immunosuppression. Cases of *Myroides* spp. infections in immunocompetent persons are uncommon, with the literature only providing three additional examples [4,5,13]. This case of *Myroides* spp. infection adds additional material to the literature for reports in immunocompetent patients, a rare occurrence in an already uncommon infection. Empiric broad spectrum antimicrobials were initiated in three of the cases in the context of severe cellulitis. Of those not started on broad spectrum antibiotics, two cases reported initiation of cefazolin secondary to targeted cellulitis coverage while the remainder were initiated on ceftriaxone and amoxicillin/clavulanic acid with similar intent [12,13,16,17]. Following isolation and confirmed growth of *Myroides* spp. from blood cultures, sensitivity testing reflected global multidrug resistance. Isolated strains demonstrated susceptibility to fluoroquinolones, carbapenems, trimethoprim-sulfamethoxazole, higher generation cephalosporin and penicillin antibiotics. All strains were resistant to aminoglycosides. This historical resistance pattern was well documented by Holmes et al. and mirrored by the current case of which the strain was only sensitive to meropenem and ciprofloxacin of the tested antibiotics [2].

The mechanisms through which *Myroides* spp. retain their resistance to numerous antimicrobials agents with a diverse array of pharmacological activities remains poorly understood. Hu et al. synthesizes preliminary opinions gathered on the hypothesized mechanisms of *Myroides* spp. pan-resistance. It was suggested that *M. odoratum* and *M. odoratimimus* demonstrate resistance to β-lactam secondary to TUS-1 and MUS-1, chromosome-encoded β-lactamases [18]. In contrast, aminoglycoside and carbapenem resistance was also studied and considered to be plasmid-mediated, citing an instance of a *Klebsiella pneumoniae* carbapenemase (KPC) gene discovered within a *Myroides* strain. Furthermore, a more recent study by the same author predicted virulence factors present in both environmental and pathogenic strains of *M. odoratimimus* isolates. Based on the presence of *bauE* (ferric siderophore binding protein paralleled in *Acinetobacter baumannii*), capsular *LPS* (complement inhibitor found in both gram-negative and gram-positive that prevents expulsion by host cells), intracellular survival factors *katA*, *clpP*, *EF-Tu*, and *sodB*, as well as bacterial DnaK (heat-shock protein involved in cellular host attachment) found within experimental strains, Hu et al. postulated that the analogous presence of these genes are what allow virulence and antibiotic resistance to gain purchase [9]. While not definitive, these mechanistic and pathogenetic models provide valuable insight into understanding the intrinsic resistance of these as well as prospective avenues for methods to circumvent the resistance of these rare and clinically challenging infections.

## Conclusion

Infections secondary to the exceptionally enigmatic *Myroides* spp. remain poorly understood. The predisposition of and poor outcomes related to immunocompromised patients advance the necessity of exploring mechanisms for resistance as well as analogous virulence factors. This organism’s multidrug resistance and infrequently confirmed presence in clinical settings speak to the need for further study and the dependence of infectious disease providers on well documented cases.

## Declaration of Competing Interest

The author declares that there is no conflict of interest regarding the publication of this case study.

## Funding

This research did not receive any specific grant from funding agencies in the public, commercial, or not-for-profit sectors.

## CRediT authorship contribution statement

**William A. Beathard:** Writing - original draft, Writing - review & editing. **Aaron Pickering:** Supervision, Writing - review & editing. **Micah Jacobs:** Conceptualization, Writing - review & editing.
